# The genome sequence of the blue-tailed damselfly, *Ischnura elegans *(Vander Linden, 1820)

**DOI:** 10.12688/wellcomeopenres.17691.1

**Published:** 2022-02-22

**Authors:** Benjamin W. Price, Martin Winter, Stephen J. Brooks, Natural History Museum Genome Acquisition Lab, Darwin Tree of Life Barcoding collective, Wellcome Sanger Institute Tree of Life programme, Wellcome Sanger Institute Scientific Operations: DNA Pipelines collective, Tree of Life Core Informatics collective, Darwin Tree of Life Consortium

**Affiliations:** 1 Department of Life Sciences, Natural History Museum, London, UK; 2 Environment Agency, Nottingham, NG2 5BR, UK

**Keywords:** Ischnura elegans, blue-tailed damselfly, genome sequence, chromosomal, Odonata

## Abstract

We present a genome assembly from an individual female *Ischnura elegans *(the blue-tailed damselfly; Arthropoda; Insecta; Odonata; Coenagrionidae). The genome sequence is 1,723 megabases in span. The majority of the assembly (99.55%) is scaffolded into 14 chromosomal pseudomolecules, with the X sex chromosome assembled.

## Species taxonomy

Eukaryota; Metazoa; Ecdysozoa; Arthropoda; Hexapoda; Insecta; Pterygota; Palaeoptera; Odonata; Zygoptera; Coenagrionidae; Ischnura; *Ischnura elegans* (Vander Linden, 1820) (NCBI:txid197161).

## Background

*Ischnura elegans*, commonly known as the blue-tailed damselfly or common bluetail, is one of the commonest damselflies in the UK, occurring at all latitudes up to the north coast of Scotland. This species is an early coloniser of new habitats and can tolerate moderately polluted water. Larvae may be found among aquatic plants in ponds, lakes, ditches, canals and slow-flowing rivers. In most of England the species is univoltine, with a life cycle completed in one year; however, in northern latitudes and likely over much of Scotland the species is semivoltine, with a life cycle completed in two years.

Males appear dark due to their metallic black abdomen, with a bright blue segment 8 and a green or blue thorax. Females have five different colour morphs: (i) violet (violacea) which mature to be either (ii) olive-green with a brown abdomen spot (infuscans), or (iii) a blue male mimic (typica); or alternatively (iv) orange-pink with a blue abdomen spot (rufescens) which mature to be (v) brown (rufescens-obsoleta) ( Brooks & Cham, 2020).

These different morphs likely have a function in mate choice, avoidance of mating harassment and camouflage. Physico-chemical analysis has shown that their coloration is mainly due to a combination of pigments and nanospheres, and that changes in the pigment composition and the packing of the nanospheres during maturation modify their colour ( Henze *et al.*, 2019). Both sexes and the different female colour morphs show differential gene expression, with the gene expression of the male mimic morph (typica) being closest to that of the male ( Chauhan *et al.*, 2016). Within females, the gene expression between morphs becomes increasingly differentiated during sexual maturation ( Willink *et al.*, 2020).

We note the recent release of a genome assembly and annotation for *I. elegans* by Chauhan *et al.* (2021). We hope that the high-quality genome assembly described herein, generated as part of the Darwin Tree of Life project, will add to this existing resource and further aid understanding of the biology, physiology and ecology of this species.

## Genome sequence report

The genome was sequenced from one female *I. elegans* collected from Iremonger pond, Nottingham, UK (latitude 52.9354, longitude -1.1544). A total of 26-fold coverage in Pacific Biosciences single-molecule long reads and 64-fold coverage in 10X Genomics read clouds were generated. Primary assembly contigs were scaffolded with chromosome conformation Hi-C data. Manual assembly curation corrected 183 missing/misjoins and removed 25 haplotypic duplications, reducing the assembly size by 1.08% and scaffold number by 56.18% and increasing the scaffold N50 by 43.82%.

**Figure 1.**
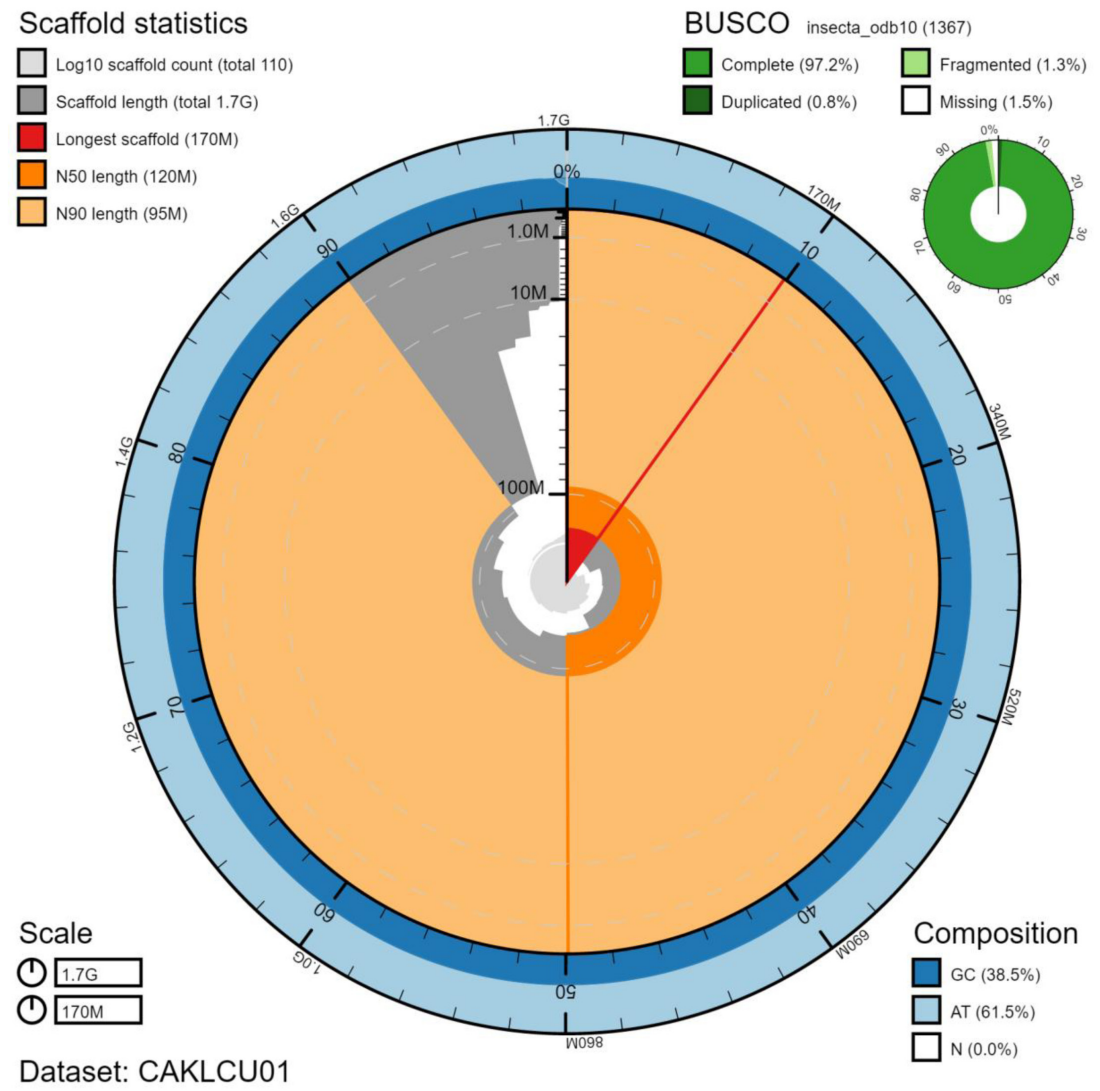
Genome assembly of *Ischnura elegans*, ioIscEleg1.1: metrics. The BlobToolKit Snailplot shows N50 metrics and BUSCO gene completeness. The main plot is divided into 1,000 size-ordered bins around the circumference with each bin representing 0.1% of the 1,722,763,890 bp assembly. The distribution of chromosome lengths is shown in dark grey with the plot radius scaled to the longest chromosome present in the assembly (170,575,982 bp, shown in red). Orange and pale-orange arcs show the N50 and N90 chromosome lengths (123,641,648 and 94,743,817 bp), respectively. The pale grey spiral shows the cumulative chromosome count on a log scale with white scale lines showing successive orders of magnitude. The blue and pale-blue area around the outside of the plot shows the distribution of GC, AT and N percentages in the same bins as the inner plot. A summary of complete, fragmented, duplicated and missing BUSCO genes in the insecta_odb10 set is shown in the top right. An interactive version of this figure is available at https://blobtoolkit.genomehubs.org/view/ioIscEleg1.1/dataset/CAKLCU01/snail.

The final assembly has a total length of 1,723 Mb in 110 sequence scaffolds with a scaffold N50 of 123.6 Mb ( Table 1). The majority of the assembly sequence (99.55%) was assigned to 14 chromosomal-level scaffolds, representing 13 autosomes (numbered by sequence length), and the X sex chromosome ( Figure 1– Figure 4; Table 2). The assembly has a BUSCO v5.1.2 ( Manni *et al.*, 2021) completeness of 97.2% (single 96.4%, duplicated 0.8%) using the insecta_odb10 reference set (n=1,367). While not fully phased, the assembly deposited is of one haplotype. Contigs corresponding to the second haplotype have also been deposited.

**Table 1.** Genome data for *Ischnura elegans*, ioIscEleg1.1.

*Project accession data*	
Assembly identifier	ioIscEleg1.1
Species	*Ischnura elegans*
Specimen	ioIscEleg1
NCBI taxonomy ID	NCBI:txid197161
BioProject	PRJEB46304
BioSample ID	SAMEA7521125
Isolate information	Female, whole organism
*Raw data accessions*	
PacificBiosciences SEQUEL II	ERR6907825, ERR6939230
10X Genomics Illumina	ERR6688441-ERR6688448
Hi-C Illumina	ERR6688440
*Genome assembly*	
Assembly accession	GCA_921293095.1
Accession of alternate haplotype	GCA_921293115.1
Span (Mb)	1,723
Number of contigs	359
Contig N50 length (Mb)	13.1
Number of scaffolds	110
Scaffold N50 length (Mb)	123.6
Longest scaffold (Mb)	170.6
BUSCO * genome score	C:97.2%[S:96.4%,D:0.8%], F:1.3%,M:1.5%,n:1367

*BUSCO scores based on the insecta_odb10 BUSCO set using v5.1.2. C= complete [S= single copy, D=duplicated], F=fragmented, M=missing, n=number of orthologues in comparison. A full set of BUSCO scores is available at https://blobtoolkit.genomehubs.org/view/ioIscEleg1.1/dataset/CAKLCU01/busco.

**Figure 2.**
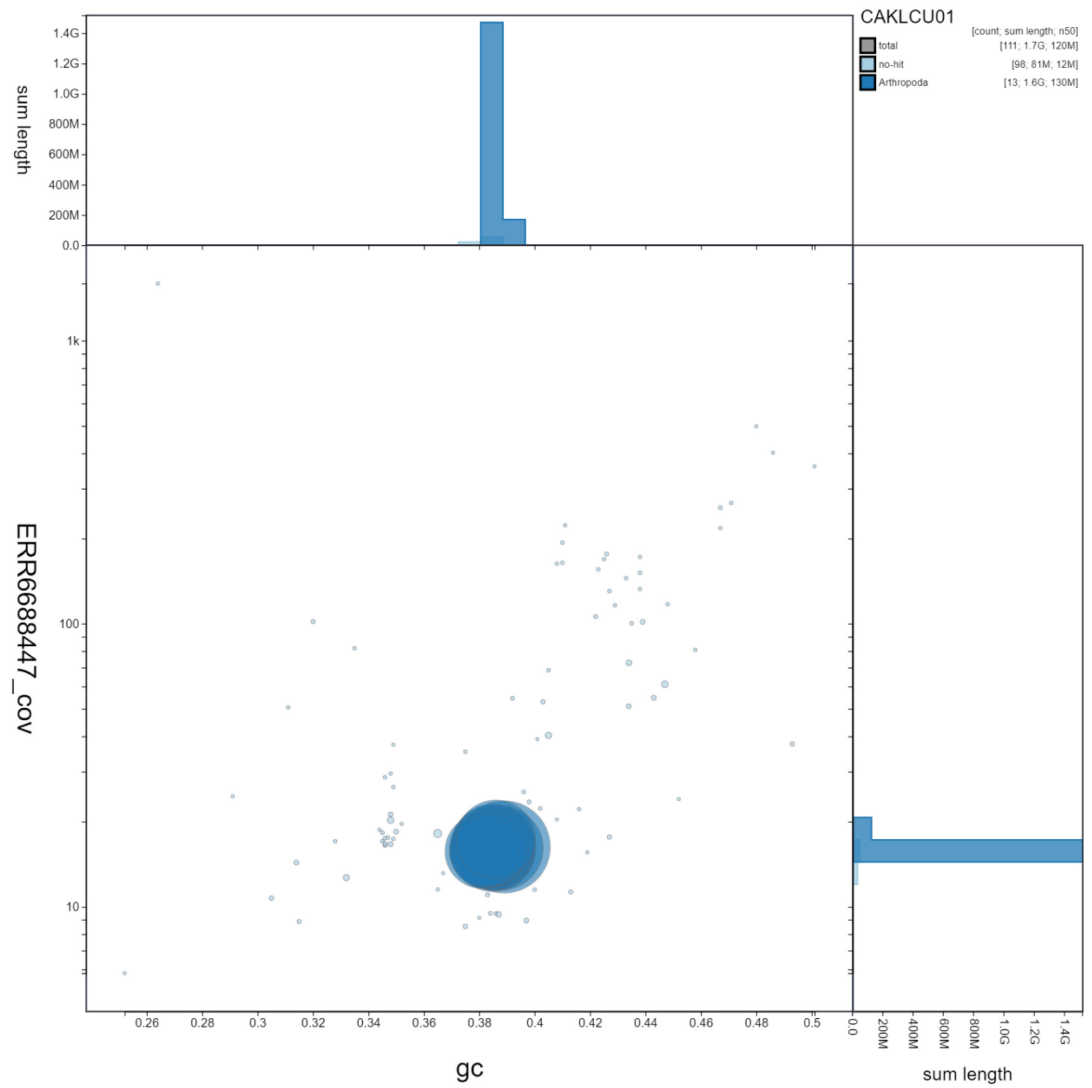
Genome assembly of *Ischnura elegans*, ioIscEleg1.1: GC coverage. BlobToolKit GC-coverage plot. Scaffolds are coloured by phylum. Circles are sized in proportion to scaffold length Histograms show the distribution of scaffold length sum along each axis. An interactive version of this figure is available at https://blobtoolkit.genomehubs.org/view/ioIscEleg1.1/dataset/CAKLCU01/blob.

**Figure 3.**
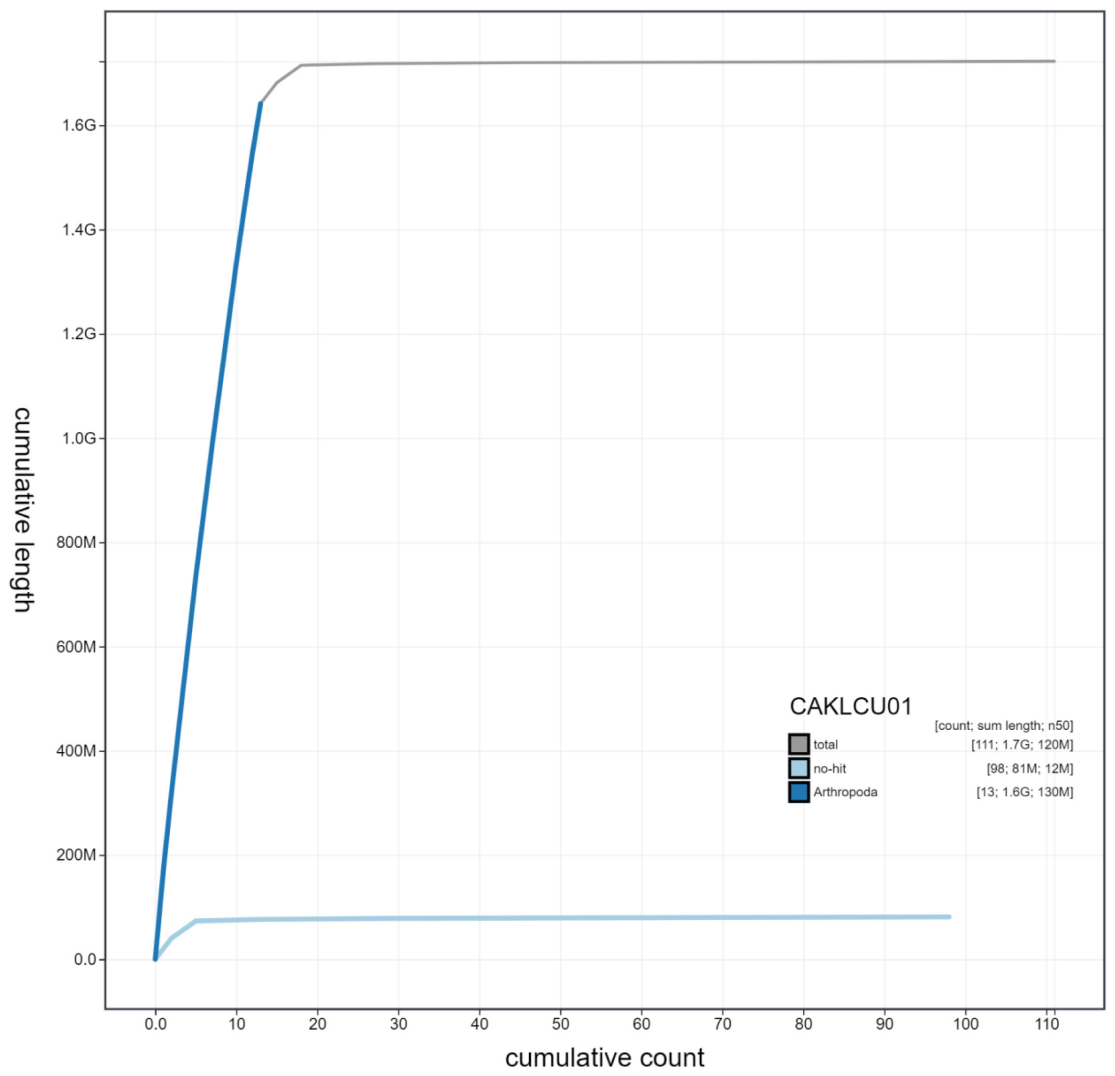
Genome assembly of *Ischnura elegans*, ioIscEleg1.1: cumulative sequence. BlobToolKit cumulative sequence plot. The grey line shows cumulative length for all scaffolds. Coloured lines show cumulative lengths of scaffolds assigned to each phylum using the buscogenes taxrule. An interactive version of this figure is available at https://blobtoolkit.genomehubs.org/view/ioIscEleg1.1/dataset/CAKLCU01/cumulative.

**Figure 4.**
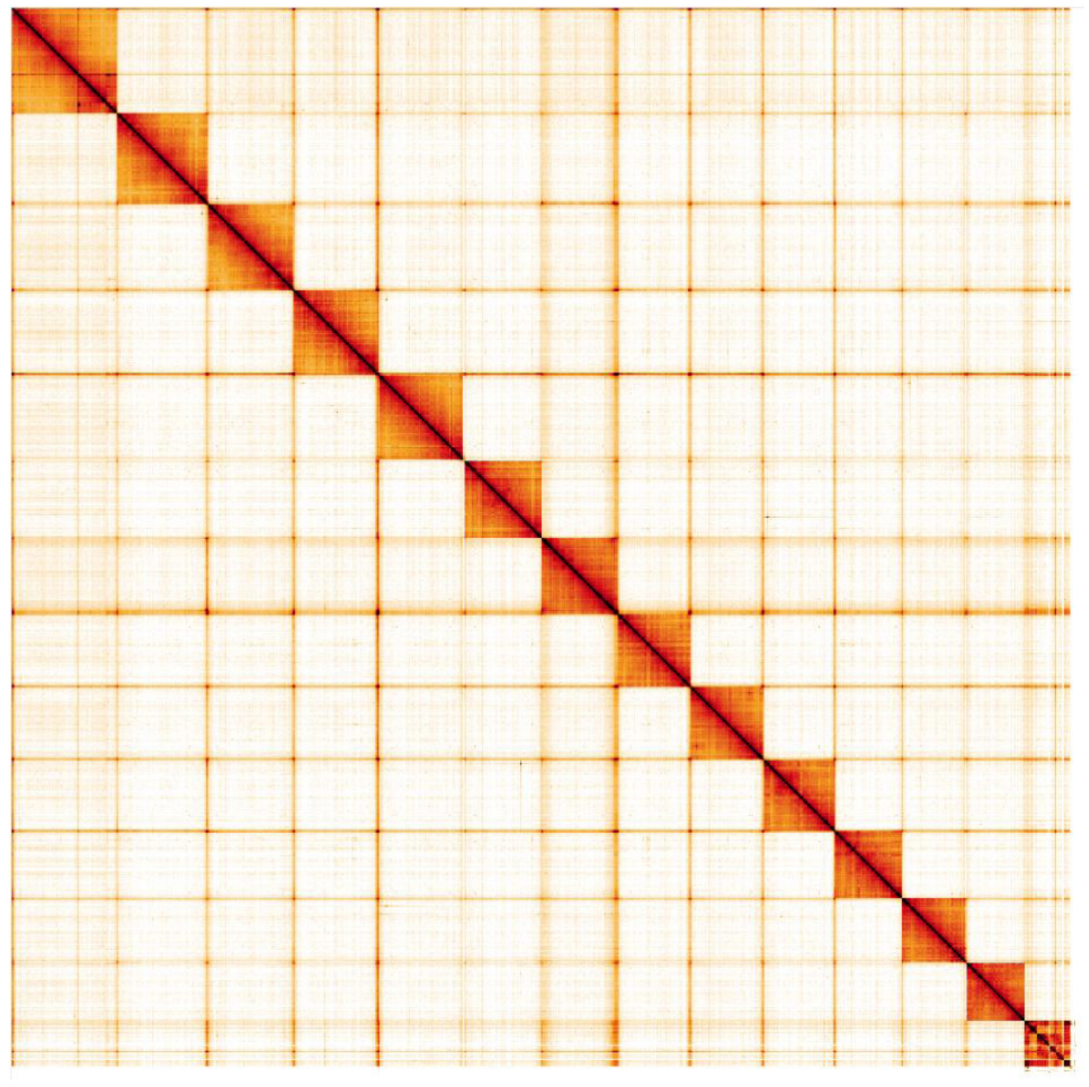
Genome assembly of *Ischnura elegans*, ioIscEleg1.1: Hi-C contact map. Hi-C contact map of the ioIscEleg1.1 assembly, visualised in HiGlass. Chromosomes are shown in order of size from left to right and top to bottom.

**Table 2.** Chromosomal pseudomolecules in the genome assembly of *Ischnura elegans*, ioIscEleg1.1.

INSDC accession	Chromosome	Size (Mb)	GC%
OV121100.1	1	170.58	38.9
OV121101.1	2	148.00	38.5
OV121102.1	3	139.04	38.8
OV121103.1	4	138.07	38.5
OV121104.1	5	137.53	38.5
OV121105.1	6	126.00	38.5
OV121107.1	7	118.30	38.5
OV121108.1	8	118.12	38.4
OV121109.1	9	115.52	38.3
OV121110.1	10	108.62	38.1
OV121111.1	11	103.41	38.4
OV121112.1	12	94.74	38.2
OV121113.1	13	21.32	38.0
OV121106.1	X	123.64	38.6
OV121114.1	MT	0.03	26.4
-	Unplaced	59.84	38.4

## Methods

### Sample acquisition and DNA extraction

A single female *I. elegans* (ioIscEleg1) was collected from Iremonger pond, Nottingham, UK (latitude 52.9354, longitude -1.1544) by Martin Winter, UK Environment Agency, using a kick-net. The sample was identified by the same individual and snap-frozen in liquid nitrogen. Unfortunately, no images were taken of the sequenced specimen during collection.

DNA was extracted at the Tree of Life laboratory, Wellcome Sanger Institute. The ioIscEleg1 sample was weighed and dissected on dry ice with tissue set aside for Hi-C sequencing. Whole organism tissue was cryogenically disrupted to a fine powder using a Covaris cryoPREP Automated Dry Pulveriser, receiving multiple impacts. Fragment size analysis of 0.01–0.5 ng of DNA was then performed using an Agilent FemtoPulse. High molecular weight (HMW) DNA was extracted using the Qiagen MagAttract HMW DNA extraction kit. Low molecular weight DNA was removed from a 200-ng aliquot of extracted DNA using 0.8X AMpure XP purification kit prior to 10X Chromium sequencing; a minimum of 50 ng DNA was submitted for 10X sequencing. HMW DNA was sheared into an average fragment size between 12–20 kb in a Megaruptor 3 system with speed setting 30. Sheared DNA was purified by solid-phase reversible immobilisation using AMPure PB beads with a 1.8X ratio of beads to sample to remove the shorter fragments and concentrate the DNA sample. The concentration of the sheared and purified DNA was assessed using a Nanodrop spectrophotometer and Qubit Fluorometer and Qubit dsDNA High Sensitivity Assay kit. Fragment size distribution was evaluated by running the sample on the FemtoPulse system.

### Sequencing

Pacific Biosciences HiFi circular consensus and 10X Genomics Chromium read cloud sequencing libraries were constructed according to the manufacturers’ instructions. Sequencing was performed by the Scientific Operations core at the Wellcome Sanger Institute on Pacific Biosciences SEQUEL II (HiFi) and Illumina NovaSeq 6000 (10X) instruments. Hi-C data were generated from remaining tissue using the Arima Hi-C+ kit and sequenced on an Illumina HiSeq X instrument.

### Genome assembly

Assembly was carried out with Hifiasm ( Cheng *et al.*, 2021); haplotypic duplication was identified and removed with purge_dups ( Guan *et al.*, 2020). One round of polishing was performed by aligning 10X Genomics read data to the assembly with longranger align, calling variants with freebayes ( Garrison & Marth, 2012). The assembly was then scaffolded with Hi-C data ( Rao *et al.*, 2014) using SALSA2 ( Ghurye *et al.*, 2019). The assembly was checked for contamination as described previously ( Howe *et al.*, 2021). Manual curation ( Howe *et al.*, 2021) was performed using HiGlass ( Kerpedjiev *et al.*, 2018) and Pretext. The mitochondrial genome was assembled using MitoHiFi ( Uliano-Silva *et al.*, 2021), which performs annotation using MitoFinder ( Allio *et al.*, 2020). The genome was analysed and BUSCO scores generated within the BlobToolKit environment ( Challis *et al.*, 2020). Table 3 contains a list of all software tool versions used, where appropriate.

**Table 3.** Software tools used.

Software tool	Version	Source
Hifiasm	0.14-r312	( Cheng *et al.*, 2021)
purge_dups	1.2.3	Guan *et al.*, 2020
SALSA2	2.2	Ghurye *et al.*, 2019
longranger align	2.2.2	https://support.10xgenomics.com/ genome-exome/software/pipelines/ latest/advanced/other-pipelines
freebayes	1.3.1-17- gaa2ace8	Garrison & Marth, 2012
MitoHiFi	2.0	( Uliano-Silva *et al.*, 2021)
HiGlass	1.11.6	( Kerpedjiev *et al.*, 2018)
PretextView	0.2.x	https://github.com/wtsi-hpag/ PretextView
BlobToolKit	2.6.4	Challis *et al.*, 2020

### Ethics/compliance issues

The materials that have contributed to this genome note have been supplied by a Darwin Tree of Life Partner. The submission of materials by a Darwin Tree of Life Partner is subject to the Darwin Tree of Life Project Sampling Code of Practice. By agreeing with and signing up to the Sampling Code of Practice, the Darwin Tree of Life Partner agrees they will meet the legal and ethical requirements and standards set out within this document in respect of all samples acquired for, and supplied to, the Darwin Tree of Life Project. Each transfer of samples is further undertaken according to a Research Collaboration Agreement or Material Transfer Agreement entered into by the Darwin Tree of Life Partner, Genome Research Limited (operating as the Wellcome Sanger Institute), and in some circumstances other Darwin Tree of Life collaborators.

## Data availability

European Nucleotide Archive: Ischnura elegans. Accession number PRJEB46304; https://identifiers.org/ena.embl/PRJEB46304.

The genome sequence is released openly for reuse. The *I. elegans* genome sequencing initiative is part of the Darwin Tree of Life (DToL) project. All raw sequence data and the assembly have been deposited in INSDC databases. The genome will be annotated and presented through the Ensembl pipeline at the European Bioinformatics Institute. Raw data and assembly accession identifiers are reported in Table 1.

## Author information

Members of the Natural History Museum Genome Acquisition Lab are listed here: https://doi.org/10.5281/zenodo.5746819.

Members of the Darwin Tree of Life Barcoding collective are listed here: https://doi.org/10.5281/zenodo.5744972.

Members of the Wellcome Sanger Institute Tree of Life programme are listed here: https://doi.org/10.5281/zenodo.5744840.

Members of Wellcome Sanger Institute Scientific Operations: DNA Pipelines collective are listed here: https://doi.org/10.5281/zenodo.5746904.

Members of the Tree of Life Core Informatics collective are listed here: https://doi.org/10.5281/zenodo.5743293.

Members of the Darwin Tree of Life Consortium are listed here: https://doi.org/10.5281/zenodo.5638618.
