# Supplementary material for: The genome sequence of the blue-tailed damselfly, Ischnura elegans (Vander Linden, 1820)
Source: Wellcome Open Res. 2022 Feb 22;7:66. [Version 1] doi: 10.12688/wellcomeopenres.17691.1 (PMC9975421; doi:10.12688/wellcomeopenres.17691.1)
Supplement: Peer review history

# Peer review: Reviewer response for version 1

**DOI:** 10.21956/wellcomeopenres.19573.r53318

**Published:** 2022-12-16

**Authors:** Rosa Ana Sanchez Guillen

**Affiliations:** 1 Red de Biología Evolutiva, Instituto de Ecología, A.C. (INECOL), Carretera antigua a Coatepec, El Haya, Xalapa, Veracruz C, Mexico

1. Correct species distribution.

*Ischnura elegans*, commonly known as the blue-tailed damselfly or common bluetail, it extends from Ireland to Japan (Askew, 1989), is one of the commonest damselflies in the Europe, occurring at all latitudes up to the north coast of Scotland.

*Citation:*

 Askew, R. (1989). The Dragonflies of Europe (B. H. & A. Harley, ed.). Colchester, England: Harley Books. doi: 10.1002/iroh.19890740432 ^1^

 2. Add reference.

 This species is an early coloniser of new habitats and can tolerate moderately polluted water (ADD REF).

 3. Correct voltinism and add citation.

 In most of England the species is univoltine, with a life cycle completed in one year; and in northern latitudes and likely over much of Scotland the species is semivoltine, with a life cycle completed in two years, however, in southern latitudes is bivoltine, with two life cycles completed in one year.

 4. Correct colour morphs description and citation

 Males appear dark due to their metallic black abdomen, with a bright blue segment 8 and a green or blue thorax. Females have five different colour morphs: (i) immature violet (violacea) thorax with a brown abdomen spot which mature to be (ii) olive-green with ( *infuscans*), or violet thorax with a blue abdomen spot which mature to be (iii) a blue male mimic ( *androchrome*); or alternatively (iv) immature orange-pink thorax with a blue abdomen spot which mature to be (v) ( *rufescens-obsoleta*) with brown thorax and a brown abdomen spot.

*Citations:*  

 Sánchez-Guillén, R. A., van Gossum, H., & Cordero-Rivera, A. (2005). Hybrization and the inheritance of female colour polymorphism in two ischnuran damselflies (Odonata : Coenagrionidae). Biological Journal of the Linnean Society, 85(4), 471–481. doi: 10.1111/j.1095-8312.2005.00506.x ^2^

 5. Correct colour morph functions and add citations

 Colour polymorphism has an evolutionary correlation with mating system in Ischnura (Sánchez-Guillén et al. 2020) with colour morph- specific fecundity and avoidance of mating harassment in *I. elegans* (Sánchez-Guillén et al. 2017).  

*Citations:*

 Sánchez-Guillén, R. A., Fadia-Cecarelli, S., Villalobos, F., Neupane, S., Rivas-Torres, A., Sanmartín-Villar, I., & Wellenreuther, M., Bybee, Seth M, Velasquez Velez, Maria Isabel, Realpe, E., J.R., Chavez-Ríos, Dumont, Henri J. & Cordero-Rivera, A. (2020). The evolutionary history of colour polymorphism in ischnuran damselflies. *International Journal of Odonatology*, *49*((3/4)), 333–370. ^3^

 Sánchez-Guillén, R. A., Wellenreuther, M., Chávez-Ríos, J. R., Beatty, C. D., Rivas-Torres, A., Velasquez-Velez, M., & Cordero-Rivera, A. (2017). Alternative reproductive strategies and the maintenance of female color polymorphism in damselflies. Ecology and Evolution, 7(15), 5592–5602. doi: 10.1002/ece3.3083 ^4^

Are sufficient details of methods and materials provided to allow replication by others?

Yes

Is the rationale for creating the dataset(s) clearly described?

Yes

Are the datasets clearly presented in a useable and accessible format?

Yes

Are the protocols appropriate and is the work technically sound?

Yes

Reviewer Expertise:

Colour polymorphism and hybridization in damselflies. Model species Ischnura elegans and I. graellsii

I confirm that I have read this submission and believe that I have an appropriate level of expertise to confirm that it is of an acceptable scientific standard.

## References

[1] : R. R. Askew: The Dragonflies of Europe. —291 pp., 502 figs., 29 colour plates. Colchester, England: Harley Books (B. H. & A. Harley Ltd.) 1988. ISBX 0-946589-100. £ 49.95 net. *Internationale Revue der gesamten Hydrobiologie und Hydrographie* .1989;74(4) : 10.1002/iroh.19890740432 457-458 10.1002/iroh.19890740432

[2] : Hybridization and the inheritance of female colour polymorphism in two ischnurid damselflies (Odonata: Coenagrionidae). *Biological Journal of the Linnean Society* .2005;85(4) : 10.1111/j.1095-8312.2005.00506.x 471-481 10.1111/j.1095-8312.2005.00506.x

[3] : The evolutionary history of colour polymorphism in Ischnura damselflies. *Odonatologica* .2020;49(3-4) :333-370 10.1111/jeb.1328929746704

[4] : Alternative reproductive strategies and the maintenance of female color polymorphism in damselflies. *Ecology and Evolution* .2017;7(15) : 10.1002/ece3.3083 5592-5602 10.1002/ece3.3083 PMC555290328811877

# Peer review: Reviewer response for version 1

**DOI:** 10.21956/wellcomeopenres.19573.r53317

**Published:** 2022-12-05

**Authors:** Panagiotis Ioannidis

**Affiliations:** 1 Institute of Molecular Biology & Biotechnology, Foundation for Research & Technology Hellas, Heraklion, Crete, Greece

The present manuscript describes the sequencing and assembly of *Ischnura elegans.*

 Since no other chromosome-level assembly is currently publicly available, the importance of this work is significant for anyone studying Palaeoptera, damselflies, or the evolution of insects, in general.

 The methodology followed by the authors is clearly written in a way that their analysis can easily be replicated by others. Also, the raw data used are deposited in SRA.

 I would very much like it, if the authors had also generated a gene set for this insect, but I guess that this is outside the scope of such manuscripts.

Are sufficient details of methods and materials provided to allow replication by others?

Yes

Is the rationale for creating the dataset(s) clearly described?

Yes

Are the datasets clearly presented in a useable and accessible format?

Yes

Are the protocols appropriate and is the work technically sound?

Yes

Reviewer Expertise:

Insect genomics and transcriptomics

I confirm that I have read this submission and believe that I have an appropriate level of expertise to confirm that it is of an acceptable scientific standard.
